# pH-Responsive Pesticide-Loaded Hollow Mesoporous Silica Nanoparticles with ZnO Quantum Dots as a Gatekeeper for Control of Rice Blast Disease

**DOI:** 10.3390/ma17061344

**Published:** 2024-03-14

**Authors:** Yi Zhao, Yanning Zhang, Yilin Yan, Zunyao Huang, Yuting Zhang, Xiaoli Wang, Nandi Zhou

**Affiliations:** The Key Laboratory of Carbohydrate Chemistry and Biotechnology, Ministry of Education, School of Biotechnology, Jiangnan University, Wuxi 214122, Chinayutingzhang@jiangnan.edu.cn (Y.Z.)

**Keywords:** nanopesticide, hollow mesoporous silica, stimuli responsive, *Magnaporthe oryzae*, prochloraz

## Abstract

Nanotechnology-enabled pesticide delivery systems have been widely studied and show great prospects in modern agriculture. Nanodelivery systems not only achieve the controlled release of agrochemicals but also possess many unique characteristics. This study presents the development of a pH-responsive pesticide nanoformulation utilizing hollow mesoporous silica nanoparticles (HMSNs) as a nanocarrier. The nanocarrier was loaded with the photosensitive pesticide prochloraz (Pro) and then combined with ZnO quantum dots (ZnO QDs) through electrostatic interactions. ZnO QDs serve as both the pH-responsive gatekeeper and the enhancer of the pesticide. The results demonstrate that the prepared nanopesticide exhibits high loading efficiency (24.96%) for Pro. Compared with Pro technical, the degradation rate of Pro loaded in HMSNs@Pro@ZnO QDs was reduced by 26.4% after 24 h ultraviolet (UV) exposure, indicating clearly improved photostability. In a weak acidic environment (pH 5.0), the accumulated release of the nanopesticide after 48 h was 2.67-fold higher than that in a neutral environment. This indicates the excellent pH-responsive characteristic of the nanopesticide. The tracking experiments revealed that HMSNs can be absorbed by rice leaves and subsequently transported to other tissues, indicating their potential for effective systemic distribution and targeted delivery. Furthermore, the bioactivity assays confirmed the fungicidal efficacy of the nanopesticide against rice blast disease. Therefore, the constructed nanopesticide holds great prospect in nanoenabled agriculture, offering a novel strategy to enhance pesticide utilization.

## 1. Introduction

Rice (*Oryza sativa* L.) is the foremost staple food crop globally. It has been estimated that over half of the global population relies on rice as their primary food source [1,2]. Rice blast disease is a fungal disease caused by *Magnaporthe oryzae* (*M. oryzae*) and is known as one of the three major diseases of rice [3,4]. The infection of rice plants with *M. oryzae* leads to lowered local pH levels in plant tissues [5,6]. Currently, the application of pesticides is still regarded as the most convenient and effective way to control fungal diseases [7]. However, the utilization of pesticides is generally poor, not only due to their loss in washing, evaporation and drifting but also due to their degradation by light, temperature and microorganisms [8,9]. Prochloraz (Pro) is a highly effective and broad-spectrum imidazole fungicide, which mainly acts by inhibiting the synthesis of ergosterol in pathogenic fungus, and it has been widely used for the control of rice blast disease [10,11]. However, as a non-systemic fungicide, the uptake of Pro by plants is limited, resulting in low utilization efficiency [12]. Therefore, it remains an urgent need to enhance the stability of pesticides in the environment and promote the uptake and transportation of pesticides within plants [13,14].

In recent years, nanotechnology has rapidly been developed and applied in the field of agriculture [15]. In 2019, nanopesticides were ranked as the first of the top ten emerging technologies in chemistry that will change the world [16]. Nanopesticides are generally defined as plant protection products with particle sizes smaller than 1000 nm, or those that bear the prefix “nano” or that exhibit new characteristics associated with small particle size [17,18]. As efficient carriers for loading pesticides, nanomaterials (NMs) can not only protect the active ingredients of pesticides from the influence of high temperatures and ultraviolet (UV) light in natural environments but also improve the chemical stability and water dispersibility of pesticides so as to promote the utilization of pesticides [19]. So far, a lot of NMs have been used as pesticide delivery carriers, including ZnO NMs, Si NMs, Cu NMs, CaCO_3_, Mg(OH)_2_ and graphene oxide (GO) [20,21]. In particular, hollow mesoporous silica nanoparticles (HMSNs) are considered to be a pesticide nanocarrier with great application prospects, because they are easy to prepare and modify, and have a large specific surface area, high loading rate and good stability [22,23].

In order to avoid leakage of the loadings in nanocarriers, some materials have been used as gatekeepers, including macrocyclic compounds, polymers, inorganic nanoparticles and biomolecules, so as to construct a variety of stimulus-responsive drug release systems in response to pH, light, temperature, redox levels, biomolecules, etc. [24,25]. For example, Gao [26] et al. constructed a pH-responsive nanosystem with disulfide bond-bridged mesoporous organosilica nanoparticles as a porous carrier and calcium carbonate as a gatekeeper to deliver Pro for the control of Sclerotinia disease. In the prepared Pro mesoporous organosilica nanoparticles, calcium was able to not only effectively improve the photostability of Pro but also respond to biological stimuli and realize the intelligent release of Pro. Kaziem [27] et al. prepared an enzyme-responsive nanopesticide by attaching α-cyclodextrin as a gatekeeper on the surface of HMSNs. The results showed that the nanopesticide not only responded to amylase in the insects’ guts but also had remarkable persistence.

ZnO quantum dots (ZnO QDs) have attracted extensive attention due to their ease of preparation, high biocompatibility and efficient ultraviolet (UV) absorption [28,29]. In addition, ZnO QDs are stable in neutral environments but readily decomposed to release Zn^2+^ in acidic environments [30,31]. Therefore, they are considered an ideal candidate for a pH-stimuli-responsive gatekeeper. Wang [32] et al. prepared a doxorubicin (DOX) delivery system that responded to dual stimuli of redox and pH by attaching ZnO QDs to the surface of mesoporous silica as capping agents via amide bonds. The acidic dissolution of ZnO QDs and the release of DOX were demonstrated through in vitro release experiments. Furthermore, based on their excellent antimicrobial properties, ZnO QDs can be utilized as an effective inorganic antimicrobial agent. Zhao [33] et al. achieved water purification by ZnO QDs@calcium alginate composite, where highly antimicrobial ZnO QDs acted as bactericides and calcium alginate was responsible for the removal of organic impurities. Qiu [34] et al. treated *M. oryzae* with ZnO nanoparticles (NPs) with an average particle size of approximately 30 nm and revealed that ZnO NPs exhibited direct antifungal activity by effectively inhibiting the formation of conidiation and appressoria. Liang [35] et al. developed a pH-responsive pesticide delivery system by conjugating ZnO QDs with kasugamycin (KAS) and evaluated the release of KAS-ZnO QDs in an acidic environment, indicating that KAS and Zn^2+^ exhibited excellent synergistic antibacterial activity against bacterial fruit blotch. However, the application of ZnO QDs as a stimuli-responsive element, a pesticide enhancer and a UV-protecting agent in smart nanodelivery systems to control rice blast disease has not been reported.

**Scheme 1:**
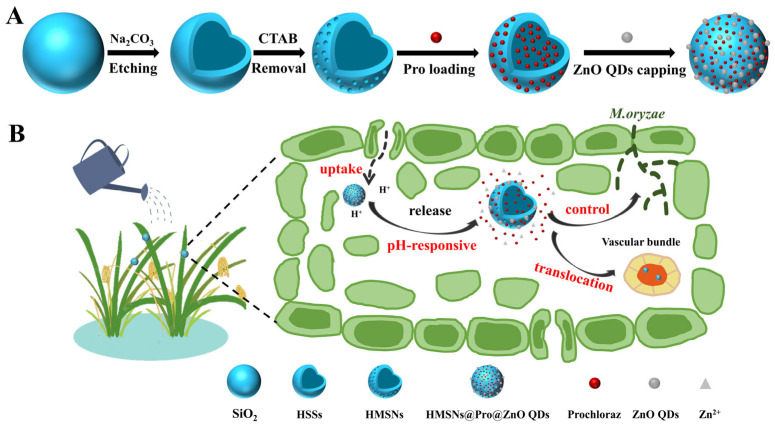
Schematic illustration of (**A**) the synthesis of HMSNs@Pro@ZnO QDs and (**B**) their application in the smart control of rice blast disease.

In this study, a pH-responsive nanopesticide delivery system is developed to prevent premature pesticide degradation, enhance fungicidal activity and improve utilization efficiency. HMSNs were used as the nanocarrier to load photosensitive pesticide Pro. Subsequently, a novel nanopesticide delivery system was fabricated via electrostatic interactions between negatively charged Pro-loaded HMSNs (HMSNs@Pro) and positively charged ZnO QDs, namely HMSNs@Pro@ZnO QDs (Scheme 1). To our knowledge, it is the first nanodelivery system where ZnO QDs are simultaneously used as a pH gatekeeper, a synergistic antimicrobial agent and a UV-protecting agent for the control of the rice blast disease.

## 2. Materials and Methods

### 2.1. Materials and Regents

Tetraethylorthosilicate (TEOS, 99%), fluorescein isothiocyanate isomer I (FITC, 90%) and Pro technical (97.0%, purity) were obtained from Shanghai Macklin Biochemical Co., Ltd. (Shanghai, China). Triethanolamine (TEA), KOH, Na_2_CO_3_, HCl (37%), NH_3_·H_2_O (14.84 M), 3-triethoxysilylpropylamine (APTE, 98%) and Potato Dextrose Agar (PDA) medium were procured from Shanghai Sinopharm Chemical Reagent Co., Ltd. (Shanghai, China). Cetyltrimethylammonium bromide (CTAB) was acquired from Shanghai Sangon Biotech Co., Ltd. (Shanghai, China). Dialysis bag (molecular weight cutoff 7 kDa) was purchased from Shanghai Greenbird Technology Development Co., Ltd. (Shanghai, China). Potato Dextrose Broth (PDB) medium was purchased from Shanghai Yuanye Bio-Technology Co., Ltd. (Shanghai, China). *M. oryzae* strain 22-586 and rice seeds were obtained from Jiangsu Academy of Agricultural Sciences. Ultrapure water was prepared by OKP-S210 ultra-low organic type laboratory ultrapure water apparatus(Shanghai Laike Industrial development Co., LTD, Shanghai, China). Analytical-grade chemicals were used for all other reagents, without additional purification.

### 2.2. Synthesis and Modification of HMSNs

HMSNs were synthesized according to the published method with some modifications [36,37]. Specifically, 1.6 mL NH_3_·H_2_O was mixed with 71.4 mL ethanol and 10 mL water, and then stirred at room temperature for 10 min. Subsequently, 2 mL TEOS was added and reacted for 1 h. Afterward, the produced SiO_2_ was obtained by centrifugation (10,000 rpm, 15 min), and then washed twice with ethanol and water, respectively, before being resuspended in 40 mL ultrapure water.

Next, 1.5 g CTAB, 64.5 mL water and 53.4 μL TEA were blended and stirred at 80 °C to ensure that CTAB was completely dissolved. Then, 30 mL the above prepared SiO_2_ was added into the mixture and stirred for 20 min, followed by addition of 450 μL TEOS and further stirring for 1 h. In order to etch the hollow structure, the solution was cooled to 50 °C, and 9 mL saturated Na_2_CO_3_ was added and incubated for 2 h. The prepared hollow silica spheres (HSSs) were centrifuged (10,000 rpm, 10 min) and washed with ethanol for three times. The above products were dispersed in a mixture of 108 mL ethanol and 12 mL HCl, and stirred at 60 °C for 12 h. HMSNs were obtained after centrifugation (10,000 rpm, 10 min) and then washed twice with ethanol and ultrapure water, respectively.

To obtain fluorescently labeled HMSNs, we performed amino-functionalization on HMSNs. The amino groups were employed to react with the isothiocyanate groups (N=C=S) of FITC molecules, leading to the formation of urea bonds and resulting in the production of HMSNs@FITC. The synthesis of HMSNs@FITC was carried out according to the reported method with some modifications [38,39]. An amount of 30 mg HMSNs was dissolved in 30 mL ethanol, and 15 μL APTE was added, followed by stirring for 24 h at room temperature. After the reaction, the solution was centrifuged (10,000 rpm, 10 min) and the supernatant was discarded. The obtained HMSNs-NH_2_ were washed with ethanol for three times, and then re-dispersed in 30 mL ethanol. An amount of 1 mg FITC was accurately weighed and added to the above suspension, which was continuously stirred for 24 h in dark. HMSNs@FITC were obtained through centrifugation (10,000 rpm, 10 min) and washing with ultrapure water for three times.

### 2.3. Synthesis of ZnO QDs

Synthesis of ZnO QDs was carried out as reported with some modifications [40]. An amount of 1.67 g Zn(Ac)_2_·2H_2_O in 50 mL ethanol was subjected to continuous stirring at 50 °C for 30 min, while 0.6 g KOH was dissolved in 10 mL ethanol and agitated with ultrasonic stirring at room temperature until complete dissolution. The KOH solution was then cautiously introduced to the Zn(Ac)_2_·2H_2_O solution, resulting in a progressive clarification of the combined mixture under the continuous stirring for 30 min. Then, 60 μL APTE mixed with 1 mL ultrapure water was added to the clarified solution. The reaction was allowed to continue for 1 h to ensure the complete hydrolysis of APTE. Finally, the solution was centrifuged (6000 rpm, 5 min) and the precipitate was washed with ethanol for three times to obtain ZnO QDs.

### 2.4. Preparation of ZnO QDs Capped HMSNs@Pro

Pro was loaded by immersion method according to the procedure described in the literature [41]. An amount of 30 mg HMSNs was added to 7.5 mL of 50 mg/mL Pro ethanol solution, and then shaken at 25 °C, 220 rpm for 12 h. HMSNs loaded with Pro were centrifuged (10,000 rpm, 10 min) and designated as HMSNs@Pro.

HMSNs@Pro and ZnO QDs were linked through electrostatic interactions. Amounts of 30 mg HMSNs@Pro and 30 mg ZnO QDs were introduced into 15 mL water and sonicated for 15 min. The produced HMSNs@Pro@ZnO QDs were collected by centrifugation (10,000 rpm, 10 min).

### 2.5. Characterization of NPs

The morphology of the NPs was observed by transmission electron microscope (TEM) (Hitachi, Ibaraki, Japan). X-ray diffraction (XRD) patterns were obtained in the range of 10°–80° on an X-ray diffractometer (Bruker, Karlsruhe, Germany). The size and zeta potential of NPs were determined according to the dynamic light scattering by a nanoparticle analyzer (Malvern, Malvern, UK). The functional groups of the NPs were determined by an infrared spectrometer (Nicolet, Madison, WI, USA).

### 2.6. Evaluation of Pro Loading

The content of Pro in HMSNs@Pro@ZnO QDs was determined by high performance liquid chromatography (HPLC) [42]. An amount of 2 mg HMSNs@Pro@ZnO QDs was dispersed in 1 mL ethanol (pH 5.0) and ultrasonicated for 3 h. Then, the supernatant was pooled through centrifugation. The determination of Pro loading in HMSNs@Pro was carried out according to the same method. HPLC (Agilent Corp., Santa Clara, CA, USA) equipped with a ZORBAX C18 reversed-phase column (4.6 × 250 mm, 5 μm) and a UV detector was used to analyze the concentration of Pro. The mobile phase was consisted of water (0.1% acetic acid) and acetonitrile (*v*/*v*, 30:70). The flow rate was set at 1.0 mL/min. The detection was performed at 220 nm. The content of Pro loading was calculated through Equation (1).

(1) $$Drug\;loading\;concent\;\left(\%\right)=\frac{weight\;of\;drug\;in\;nanoparticles}{weight\;of\;nanoparticles\;taken}\times 100\%$$

### 2.7. Characterization of Controlled Release Behavior

In order to investigate the release behavior of HMSNs@Pro@ZnO QDs, PBS buffers containing 0.5% tween-80 with different pH (5.0, 6.5, 7.4) were prepared as the release media. Appropriate amount of HMSNs@Pro@ZnO QDs was mixed with 1 mL release medium and then put in the dialysis bag. The dialysis bag was sealed and submerged in 19 mL release medium, and incubated with shaking at 120 rpm. Determination of HMSNs@Pro was carried out with the same procedure.

At the certain time intervals, 1 mL release medium was sampled and 1 mL fresh medium was added immediately to ensure the volume to be constant. And the cumulative release at different time was calculated according to Equation (2), where M_t_ represents the mass of Pro released from the nanoparticles at each sampling time point, and M_0_ represents the total mass of Pro in the nanoparticles.

(2) $$Cumulative\;release\;rate\;(\%)=\sum_{t=0}^{t}\frac{M_{t}}{M_{0}}\times 100\%$$

### 2.8. Evaluation of In Vitro Fungicidal Activity

The in vitro fungicidal activity of HMSNs@Pro@ZnO QDs against *M. oryzae* was investigated by detection of mycelial growth rate. Different concentrations of Pro technical or HMSNs@Pro@ZnO QDs were mixed with a certain amount of PDA medium, so that the final concentrations of the active ingredient were 0.0125, 0.025, 0.05, 0.1 and 0.2 μg/mL, respectively. Then, the activated mycelial discs (7 mm, 13 days old) of *M. oryzae* were placed in the center of the above prepared media. The average diameter of the mycelium was measured after incubation for a certain period of time at 28 °C in dark. The inhibition was calculated through Equation (3), and the EC_50_ values were calculated by SPSS.

(3) $$Inhibition\;\left(\%\right)=(1-\frac{mycelial\;diameter\;of\;treatment}{mycelial\;discs\;diameter})\times 100\%$$

### 2.9. Evaluation of HMSNs@Pro@ZnO QDs for Rice Blast Disease Control 

The control efficacy of Pro technical and HMSNs@Pro@ZnO QDs against *M. oryzae* was further verified by the detached leaf inoculation [43]. Leaves of rice seedlings cultured to the three-leaf-one-heart period were cut and placed in Petri dishes with the leaves upwards and moisturized with two layers of the filter paper. Pro technical and HMSNs@Pro@ZnO QDs were diluted to 100 μg/mL with 0.1% tween-80. A volume of 500 μL different pesticides was sprayed onto the above leaves and naturally placed to dry at room temperature. At the same time, the activated mycelial discs (3 mm, 3 days old) of *M. oryzae* were cultured in PDB medium at 28 °C, 175 rpm for 3 days. Then, the cultured mycelial balls were placed on the dried leaves and incubated at 25 °C for 48 h. The lesion area was measured, and the control efficacy was analyzed. The control efficacy was calculated through Equation (4) [44].

(4) $$Control\;efficacy\;=\left(1-\frac{the\;lesion\;area\;of\;each\;treatment}{the\;lesion\;area\;of\;CK}\right)\times 100\%$$

### 2.10. Photostability of HMSNs@Pro@ZnO QDs

To determine the photostability of the nanopesticide, HMSNs@Pro@ZnO QDs were dissolved in an appropriate amount of ethanol and then dispensed in the centrifuge tubes at a volume of 1 mL each. The samples were irradiated using a UV lamp (6 W, λ = 245 nm), with the light source positioned 10 cm away from the liquid surface in the centrifuge tubes. The centrifuge tubes with different treatment time were taken to determine the remaining Pro content by HPLC. The photolysis rate was calculated using Equation (5), where M_t_ represents the mass of Pro remaining at the sampling time point, and M_0_ represents the total mass of Pro in the nanoparticles [45]. The photostability of Pro technical was determined by the same method.

(5) $$Photolysis\;rate\;\left(\%\right)=\left(1-\frac{M_{t}}{M_{0}}\right)\times 100\%$$

### 2.11. Investigation of Distribution and Translocation of HMSNs@FITC in Plant Tissue

To verify the uptake and subsequent distribution of HMSNs in the rice plants, rice seedlings were cultured hydroponically. Then, the roots and leaves of these plants were treated with HMSNs@FITC. The uptake and subsequent distribution of HMSNs@FITC in the leaves were investigated by immersing the leaves in 0.5 mg/mL HMSNs@FITC for 5 min, at which time the roots were still incubated with the nutrient solution. In addition, to investigate the transportation of HMSNs from the roots to other parts, the roots of rice plants were incubated in 0.5 mg/mL HMSNs@FITC. The distribution of HMSNs@FITC in the tissues of the rice plants was observed by confocal laser scanning microscopy (CLSM)(Leica, Wetzlar, Germany) at an excitation wavelength of 488 nm.

## 3. Results and Discussion

### 3.1. Synthesis and Characterization of NMs

**Figure 1:**
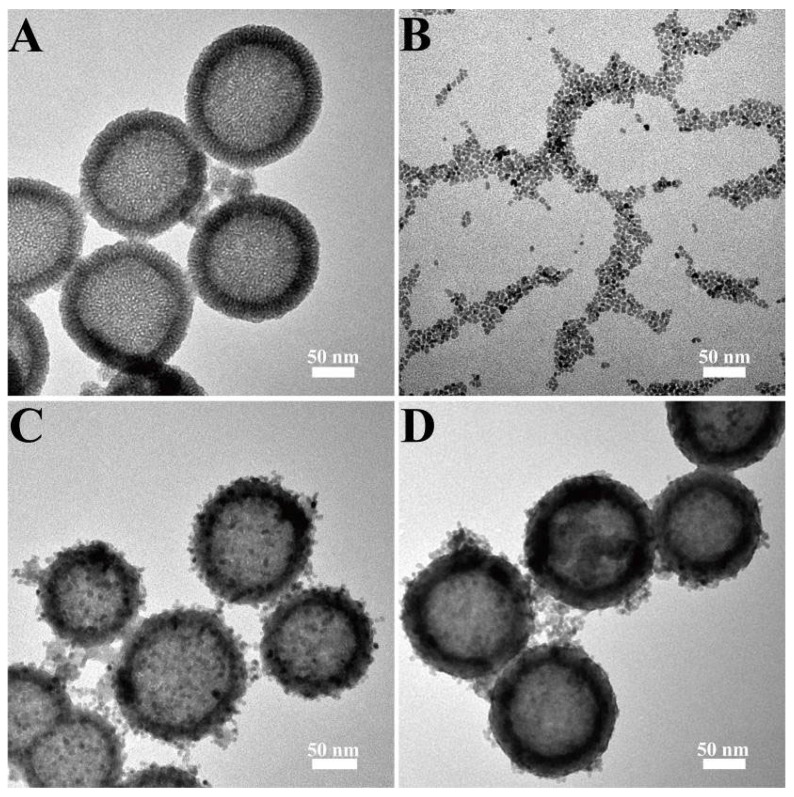
TEM images of (**A**) HMSNs, (**B**) ZnO QDs, (**C**) HMSNs@ZnO QDs and (**D**) HMSNs@Pro@ZnO QDs.

In this study, HMSNs were synthesized as a carrier through the self-template method. In brief, SiO_2_ NPs were synthesized by hydrolyzing TEOS in an alkaline environment. Subsequently, the template agent CTAB and TEOS were introduced to form the shell structure. HMSNs were finally obtained by etching silica sphere template with the saturated Na_2_CO_3_ solution and subsequent removal of CTAB. TEM was firstly employed to characterize HMSNs, ZnO QDs, HMSNs@ZnO QDs and HMSNs@Pro@ZnO QDs. As shown in Figure 1A, the synthesized HMSNs, with an average diameter of 142 nm, have the obvious hollow structure and the mesoporous structure. The particle size and morphology of HMSNs are consistent with those reported in the literature [38]. ZnO QDs with small particle size and uniform morphology were prepared by sol-gel method. As displayed in Figure 1B, the morphology of ZnO QDs is spherical. With an average particle size of 4.53 nm, ZnO QDs are well dispersed without significant aggregation. For HMSNs@ZnO QDs, it can be observed that numerous ZnO QDs are densely combined to the surface of HMSNs. Moreover, the mesoporous structure of HMSNs can no longer be observed in HMSNs@ZnO QDs and the particle size of HMSNs@ZnO QDs increases to 145.59 nm. This indicates that the mesoporous entrance of HMSNs has been successfully blocked by ZnO QDs. (Figure 1C). Simultaneously, the morphological structure of HMSNs@Pro@ZnO QDs was characterized. As depicted in Figure 1D, the hollow and mesoporous structure of HMSNs@Pro@ZnO QDs becomes further obscured due to the loading of Pro, resulting in the increased particle size of 173.94 nm. This indicates that HMSNs@Pro@ZnO QDs have been successfully prepared.

UV–vis absorption spectra of ZnO QDs are shown in Figure S1A. ZnO QDs have no absorption in the visible region, yet have a distinct characteristic band-edge absorption peak at 370 nm. ZnO QDs show fluorescence at different excitation wavelengths (360–390 nm), but the maximum excitation wavelength is 370 nm (Figure S1B). It can be clearly observed that the ZnO QDs solution exhibits a transparent color under daylight (Figure S2A), and a yellow color under UV light (Figure S2B). These phenomena indicate that ZnO QDs have been successfully synthesized.

**Figure 2:**
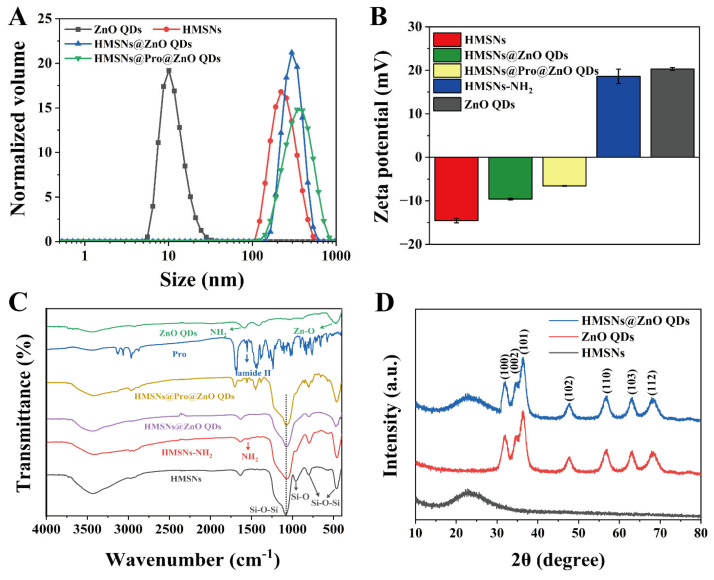
Characterization of the nanoparticles. (**A**) Hydrodynamic diameter distributions of ZnO QDs, HMSNs, HMSNs@ZnO QDs and HMSNs@Pro@ZnO QDs; (**B**) Zeta potential of HMSNs, HMSNs@ZnO QDs, HMSNs@Pro@ZnO QDs, HMSNs−NH_2_ and ZnO QDs; (**C**) FTIR spectra of HMSNs, HMSNs−NH_2_, HMSNs@ZnO QDs, HMSNs@Pro@ZnO QDs, Pro and ZnO QDs; (**D**) XRD patterns of HMSNs, ZnO QDs and HMSNs@ZnO QDs.

The synthesis of the NPs was further characterized using the hydrodynamic diameter. As shown in Figure 2A, the hydrodynamic diameters of ZnO QDs and HMSNs are 10.1 nm and 232.3 nm, respectively. Upon the combination of ZnO QDs, the hydrodynamic diameter of HMSNs increases from 232.3 nm to 299.1 nm. Moreover, the hydrodynamic diameter of HMSNs@Pro@ZnO QDs is 333.9 nm. The variation in the hydrodynamic diameter indicates the successful preparation of NPs.

Determination of the charged state of the NPs is beneficial for assessing whether the electrostatic interaction occurs. As displayed in Figure 2B, the zeta potential of HMSNs is −14.57 mV, due to the large number of silicon hydroxyl groups on their surface. When HMSNs are modified with APTE, the zeta potential of HMSNs-NH_2_ is 18.6 mV, indicating that the amino group has been successfully modified on the surface of HMSNs. ZnO QDs show a positive potential of 20.3 mV, which can be attributed to their amination modification. With the combination of ZnO QDs on the HMSNs surface, the potential of HMSNs@ZnO QDs shifts from −14.57 mV to −9.58 mV. The change in the potential is also due to the adsorption of ZnO QDs to HMSNs via the electrostatic interaction. Furthermore, following the loading of Pro, the zeta potential of HMSNs@Pro@ZnO QDs augments to −6.56 mV, thus confirming the successful synthesis of HMSNs@Pro@ZnO QDs.

By utilizing FTIR spectra, a comprehensive analysis of the chemical bonds, the functional groups and the structures of the NPs can be conducted. Therefore, FTIR spectra were further employed for characterizing the synthesized NPs. As shown in Figure 2C, the infrared spectrum of HMSNs exhibits the characteristic absorption peaks, including the asymmetric stretching vibration of Si-O-Si at 1080 cm^−1^, the stretching vibration of Si-O at 960 cm^−1^, the symmetric stretching vibration of Si-O-Si at 800 cm^−1^ and the bending vibration of Si-O-Si at 467 cm^−1^ [46]. In the infrared spectrum of HMSNs-NH_2_, a new absorption peak of -NH_2_ at 1548 cm^−1^ is observed, indicating that the amino group has been successfully modified on the surface of HMSNs. The stretching vibration of N-H at 1580 cm^−1^ and the stretching vibration of Zn-O at 473 cm^−1^ are observed in ZnO QDs, signifying the successful synthesis of APTE modified ZnO QDs. The characteristic peaks of Si-O- Si and Si-O can be observed in HMSNs@Pro@ZnO QDs. Additionally, the stretching vibrations of Pro amide II [44] and Zn-O at 473 cm^−1^ are also detected in HMSNs@Pro@ZnO QDs, confirming the successful loading of Pro and the effective combination of ZnO QDs.

XRD spectra was further used to verify the structure and purity of ZnO QDs and HMSNs@ZnO QDs (Figure 2D). HMSNs have an amorphous structure, so they show a broad peak between 20° and 30°. The characteristic peaks of ZnO QDs appear at 31.85, 34.75, 36.28, 47.67, 56.72, 62.97 and 68.25°, indicating that the prepared ZnO QDs possess the hexagonal wurtzite structure [32]. For HMSNs@ZnO QDs, both the amorphous peak of HMSNs and the characteristic peaks of ZnO QDs can be observed. Moreover, the absence of the redundant diffraction peaks indicates the high purity of the prepared NMs. All these results validate the successful synthesis of the NMs.

### 3.2. Release Performance of HMSNs@Pro and HMSNs@Pro@ZnO QDs

**Figure 3:**
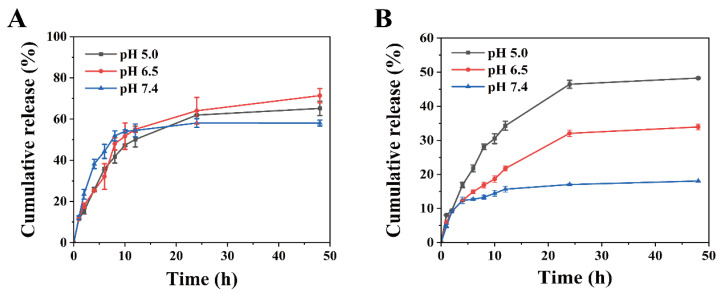
Effects of pH on the release behaviors of HMSNs@Pro (**A**) and HMSNs@Pro@ZnO QDs (**B**).

HMSNs were employed as the carrier of Pro, while ZnO QDs were used as the gatekeeper to respond to the pH in the environment and control the release of Pro. Thus, the release performance of HMSNs@Pro and HMSNs@Pro@ZnO QDs was studied and compared. The loading contents of Pro in HMSNs@Pro and HMSNs@Pro@ZnO QDs are 46.76% and 24.96%, respectively. The reduced drug loading in HMSNs@Pro@ZnO QDs may be attributed to the release of a portion of Pro under the influence of ultrasonication during the modification of ZnO QDs. The cumulative release of Pro from the two nanocarriers was tested. As shown in Figure 3A, the release manners of HMSNs@Pro are almost identical at different pH. Within the first 12 h, the release rate of Pro is relatively fast, and the release reaches the maximum by 48 h. Obviously, HMSNs@Pro cannot respond to pH. However, it can be clearly observed that the release profiles of HMSNs@Pro@ZnO QDs at different pH are significantly different (Figure 3B). ZnO QDs are readily decomposed in the acidic condition, which in turn triggers the release of Pro from HMSNs@Pro@ZnO QDs. To demonstrate the effect of pH on ZnO QDs, ZnO QDs were incubated in the solution with different pH. When the pH changes from 7.4 to 3.0, the color of the suspension gradually changes from yellow to colorless, and it can be assumed that ZnO QDs are continuously decomposed with the decrease in pH, which leads to the disappearance of the fluorescence (Figure S3). As shown in Figure 3B, the cumulative releases of Pro after 48 h are 48.26% at pH 5.0 and 33.92% at pH 6.5, respectively, while it is only 18.07% at pH 7.4. Muhammad [47] et al. presented a cancer drug DOX delivery system (ZnO QDs@MSNs-DOX) that utilizes ZnO QDs to plug nanopores in MSNs. In vitro release experiments confirmed that the rapid release of DOX at pH 5.0 was attributed to the dissolution of ZnO QDs in an acidic environment, aligning with the in vitro release outcomes in our investigation. These results indicate that the prepared nanopesticide can respond to the change in pH and realize the intelligent release of the pesticide.

The release kinetics of Pro from HMSNs@Pro and HMSNs@Pro@ZnO QDs were analyzed by the zero-order, first-order, Higuchi and Ritger–Peppas models. The zero-order model: $\frac{M_{t}}{M_{\infty }}=kt$; the first-order model: $\frac{M_{t}}{M_{\infty }}=1-e^{-kt}$; the Higuchi model: $\frac{M_{t}}{M_{\infty }}={kt}^{1/2}$ and the Ritger–Peppas model: $\frac{M_{t}}{M_{\infty }}={kt}^{n}$, where M_t_ is the amount of the released pesticide at time t, M_∞_ is the maximal amount of the released pesticide at infinite time, k is the rate constant of the pesticide, and n is the diffusion exponent. The linear regression coefficient R^2^ obtained from the fitting equations was used to assess the applicability of the fitted models (Table 1 and Tables S1 and S2). The release of both HMSNs@Pro and HMSNs@Pro@ZnO QDs at different pH conforms to the first-order model, indicating that the release rate of Pro is directly proportional to the concentration of Pro in the nanopesticide. In addition, the Ritger–Peppas release model analyzed the release behavior of the pesticides by determining the value of the diffusion exponents n [48]. In this study, the spherical HMSNs were employed as the carrier of the pesticide, thus when n ≤ 0.43, the drug release follows the Fickian diffusion; while 0.43 < n < 1.0, the drug release demonstrates non-Fickian diffusion behavior [49,50]. As shown in Tables S1 and S2, the fitted n values are all <0.43, indicating that the release of Pro is the Fickian diffusion. Due to the presence of a certain amount of externally loaded Pro during the preparation of the sustained-release system, Pro adsorbed on the surface of HMSNs exhibits a relatively fast diffusion rate in the initial stage of the release. Conversely, Pro inside HMSNs requires diffusion through the mesoporous channels, resulting in a slower diffusion rate, manifesting as a slow release phenomenon.

### 3.3. Control Efficiency of HMSNs@Pro@ZnO QDs against M. oryzae

*M. oryzae* is one of the phytopathogenic fungi that severely damages plants. Firstly, the activity of ZnO QDs against *M. oryzae* was evaluated by mycelial growth inhibition. When the effective concentration of ZnO QDs is 0.1 mg/mL, the inhibition rate of *M. oryzae* reaches 3.9%. As the effective concentration of ZnO QDs increases to 1.0 mg/mL, the inhibition rate remarkably increases to 55.84% (Figure S4). Therefore, it can be concluded that ZnO QDs have a concentration-dependent inhibition effect on the growth of *M. oryzae*. In the study conducted by Qiu [34] et al., ZnO NPs were found to have no significant inhibitory effect on the growth of the rice blast fungus mycelium; instead, they exhibited the anti-rice blast fungus activity by inhibiting the formation of conidiation and appressorium. However, this study has revealed that ZnO QDs can directly suppress the growth of the rice blast fungus mycelium.

**Figure 4:**
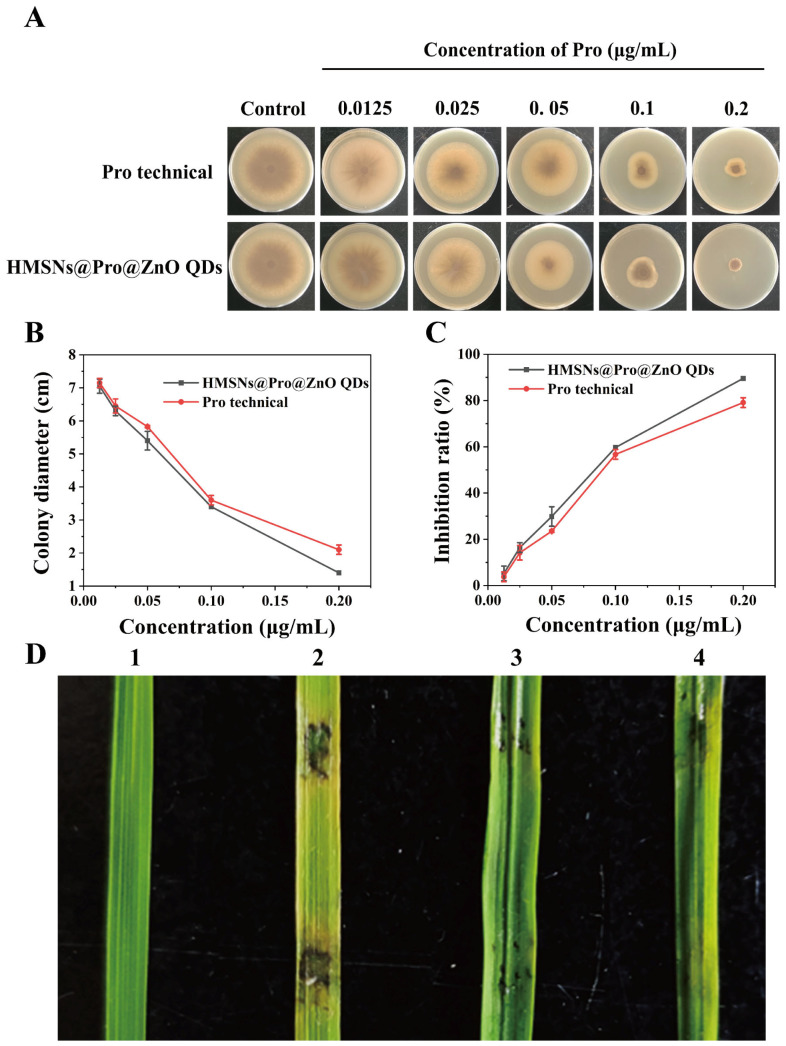
(**A**) Digital images of *M. oryzae* treated with different concentrations of Pro technical and HMSNs@Pro@ZnO QDs for 13 days; (**B**) Colony diameter and (**C**) Inhibition ratio of *M. oryzae* treated with the different concentrations of Pro technical and HMSNs@Pro@ZnO QDs for 13 days; (**D**) Images of the rice leaves treated with the different reagents and inoculated with the mycelial balls of *M. oryzae* for 48 h; (1) 500 μL of 0.1% tween-80; (2) 500 μL of 0.1% tween-80 + mycelial balls; (3) 500 μL of 100 μg/mL Pro technical + mycelial balls; (4) 500 μL of 100 μg/mL HMSNs@Pro@ZnO QDs + mycelial balls.

In order to investigate the inhibition effect of Pro technical and HMSNs@Pro@ZnO QDs on *M. oryzae*, five concentration gradients were set up for the studies. As shown in Figure 4A, it can be seen that both Pro technical and HMSNs@Pro@ZnO QDs can effectively inhibit the growth of *M. oryzae* on the PDA medium. The colony diameter of *M. oryzae* on the PDA medium decreases with the increase in the concentration of Pro (Figure 4B). The inhibition rate of Pro technical against *M. oryzae* is slightly lower than that of HMSNs@Pro@ZnO QDs with the same dosage of Pro (Figure 4C). Moreover, the EC_50_ values of Pro technical and HMSNs@Pro@ZnO QDs are 0.088 and 0.072 μg/mL, respectively (Table S3). Therefore, it can be speculated that the slightly improved inhibition of HMSNs@Pro@ZnO QDs than Pro technical is due to the synergistic inhibition of Pro and ZnO QDs.

The control efficacy of Pro technical and HMSNs@Pro@ZnO QDs against *M. oryzae* was further verified using the rice leaves. As shown in Figure 4D and Table S4, when the rice plants are inoculated with *M. oryzae* after 1 h of the pesticide spraying, both Pro technical and HMSNs@Pro@ZnO QDs show the excellent control of the rice blast disease. The leaves treated with HMSNs@Pro@ZnO QDs show the smallest lesion area and the high bioactivity, with the control efficacy of 89.4%. While the control efficacy of the leaves treated with Pro technical is 86.72%, which exhibits the insignificant difference from each other. The above results can indicate that at the same concentration, HMSNs@Pro@ZnO QDs slightly increase in the effectiveness than that of Pro technical in the control of *M. oryzae.*

### 3.4. Evaluation of the Photostability of the Pesticide

**Figure 5:**
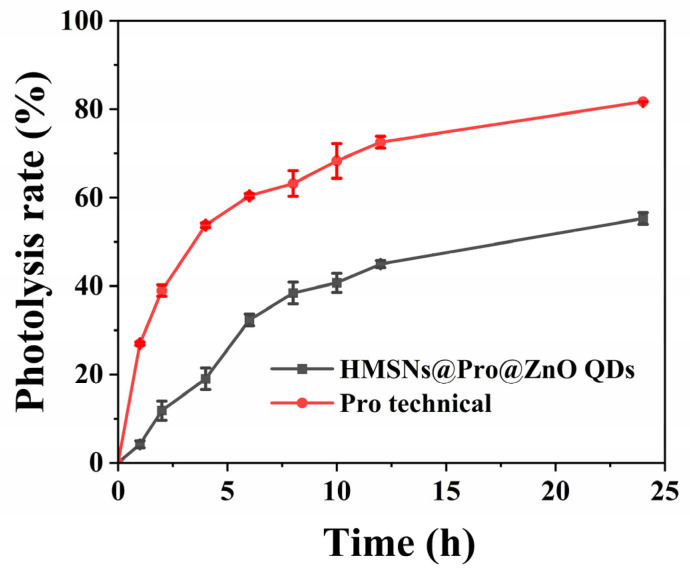
Stability of Pro technical and HMSNs@Pro@ZnO QDs under UV irradiation.

Pro is sensitive to UV light, so one of the effective ways to improve the utilization is to avoid its direct exposure to UV light. In this study, the nanocarrier is not only used as the controlled-release carrier, but also the protective agent of Pro avoiding UV degradation. Therefore, the degradation rates of Pro technical and HMSNs@Pro@ZnO QDs were determined over 24 h of UV irradiation and compared. As shown in Figure 5, the degradation rates of Pro technical and Pro loaded in HMSNs@Pro@ZnO QDs are 27.0% and 4.2%, respectively, after being irradiated by UV light for 1 h. After 24 h of UV irradiation, the degradation rate of Pro technical reaches 81.7%, at which time the degradation rate of Pro loaded in HMSNs@Pro@ZnO QDs is only 55.3%. The above results indicate that HMSNs@Pro@ZnO QDs can effectively protect Pro from degradation, which can mainly be attributed to the shielding or the absorbing ability of HMSNs and ZnO QDs for UV, thus improving the utilization of Pro at a certain extent.

### 3.5. Study on the Translocation of HMSNs@FITC in Plants

**Figure 6:**
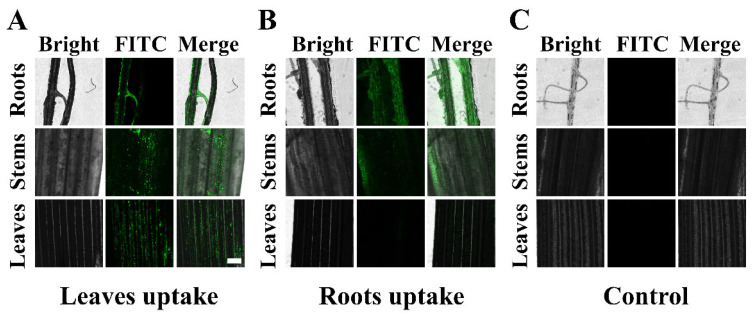
CLSM images of the tissues (roots, stems and leaves) of the rice plants after 48 h treatment with HMSNs@FITC. (**A**) Leaves uptake, (**B**) roots uptake, and (**C**) control group. Scale bar = 250 μm.

In order to investigate the uptake and subsequent distribution of HMSNs in the rice plants, FITC was attached to the surface of HMSNs. Generally, NPs can enter the plants through roots and leaves. Therefore, the roots and leaves of the plants were separately treated to observe the transportation of HMSNs from the leaves or the roots to other parts of the plants. After applying of HMSNs@FITC, the roots, stems and leaves of the plants were monitored under the confocal microscope. As shown in Figure 6A, the green fluorescence can be observed in all tissues of the leaf-treated rice plants, indicating that HMSNs@FITC can be transported to all parts of the plants through the leaves. However, the significant fluorescent signals are observed only in the roots and the stems of the root-treated rice plants. Only very low fluorescent signal can be identified in the leaves (Figure 6B) [44]. For those blank control, no fluorescent signal can be observed (Figure 6C).

It is generally believed that when the size of NPs is smaller than 4.8 nm, the NPs can directly penetrate the cuticle and enter the leaf tissues. Whereas the particle size exceeds 4.8 nm, the NPs can enter the apoplast through the stomatal pathway, and then undergo the long-distance transportation through the vascular bundles. However, due to the presence of the Casparian strip in the root, the NPs entering the plants through the root application may not be able to enter the vascular bundle for the long-distance transportation [51,52]. This can explain why HMSNs@FITC by the foliar application can be detected in the roots, stems and leaves, whereas HMSNs@FITC by the root application cannot be detected in the leaves.

## 4. Conclusions

In summary, a smart pH-responsive nanopesticide was successfully constructed through the self-assembly via the electrostatic interaction, with ZnO QDs serving as the gatekeeper and HMSNs as the carrier. The nanopesticide showed a pH-responsive controlled release of Pro to efficiently inhibit the rice blast disease. The prepared HMSNs@Pro@ZnO QDs can not only respond to the change in the microenvironment of the fungal infection site, leading to the controlled release of Pro, but also prevent Pro from degradation by shielding and absorbing UV light, which can improve its utilization in the actual agricultural production. The synergistic function of Pro and the dissolved zinc ion also slightly enhanced the inhibition effect. Moreover, in vitro and the isolated leaf experiments showed that the prepared nanopesticide possesses excellent fungicidal activity against *M. oryzae* by effectively inhibiting mycelial growth. In addition, HMSNs can be transported to various parts of the rice plants through the leaf pathway, which provides an important basis for HMSNs as a pesticide carrier into the plants to deliver the pesticides. Therefore, this smart pesticide delivery system may have great prospects in nanoenabled agriculture.

## Figures and Tables

**Table 1:** Kinetic equations for the first-order release of HMSNs@Pro and HMSNs@Pro@ZnO QDs at different pH.

Sample	Models	pH	Fitting Equation	R^2^
		5.0	y = 64.74(1−e^−0.13 x^)	0.9960
HMSNs@Pro	First order	6.5	y = 70.08(1−e^−0.13 x^)	0.9842
		7.4	y = 58.08(1−e^−0.26 x^)	0.9982
		5.0	y = 49.19(1−e^−0.10 x^)	0.9925
HMSNs@Pro@ZnO QDs	First order	6.5	y = 34.37(1−e^−0.09 x^)	0.9659
		7.4	y = 16.28(1−e^−0.32 x^)	0.9524

## Data Availability

Data are contained within this article and Supplementary Materials.

## Supplementary Materials

The following supporting information can be downloaded at: https://www.mdpi.com/article/10.3390/ma17061344/s1, Figure S1: (A) UV–vis absorption spectra of ZnO QDs at different concentrations; (B) fluorescence spectra of ZnO QDs at different excitation wavelength. Figure S2: Digital images of ZnO QDs under daylight (A) and 365 nm UV lamp (B). Figure S3: Dissolution of ZnO QDs in PBS at different pH. Figure S4: (A) Images of *M. oryzae* treated with different concentrations of ZnO QDs for 13 days. (B) Inhibition ratio of *M. oryzae* treated with different concentrations of ZnO QDs for 13 days. Table S1: Release kinetic equations of HMSNs@Pro at different pH. Table S2: Release kinetic equations of HMSNs@Pro@ZnO QDs at different pH. Table S3: Fungicidal activity of Pro technical and HMSNs@Pro@ZnO QDs against *M. oryzae*. Table S4: Effects of Pro technical, HMSN@Pro@ZnO QDs and 0.1% tween-80 on the lesion development by *M. oryzae* on rice leaves.

## References

[1] Sasaki T. (2005). The map-based sequence of the rice genome. Nature.

[2] Hashim N., Ali M.M., Mahadi M.R., Abdullah A.F., Wayayok A., Mohd Kassim M.S., Jamaluddin A. (2024). Smart Farming for Sustainable Rice Production: An Insight into Application, Challenge, and Future Prospect. Rice Sci..

[3] Dauda W.P., Singh Rana V., Solanke A.U., Krishnan G., Bashya B.M., Aggarwal R., Shanmugam V. (2022). Metabolomic analysis of sheath blight disease of rice (*Oryza sativa* L.) induced by Rhizoctonia solani phytotoxin. J. Appl. Microbiol..

[4] Motoyama T., Kondoh Y., Shimizu T., Hayashi T., Honda K., Uchida M., Osada H. (2022). Identification of Scytalone Dehydratase Inhibitors Effective against Melanin Biosynthesis Dehydratase Inhibitor-Resistant Pyricularia oryzae. J. Agric. Food Chem..

[5] Pradhan A., Ghosh S., Sahoo D., Jha G. (2021). Fungal effectors, the double edge sword of phytopathogens. Curr. Genet..

[6] Hogan D.A., Vylkova S. (2017). Environmental pH modulation by pathogenic fungi as a strategy to conquer the host. PLoS Pathog..

[7] Masudulla K., Azhar U.K., Mohd Abul H., Krishna Kumar Y., Marina M.C.P., Nazia M., Virendra Kumar Y., Afzal Husain K., Saiful I., Gulshan Kumar S. (2021). Agro-Nanotechnology as an Emerging Field: A Novel Sustainable Approach for Improving Plant Growth by Reducing Biotic Stress. Appl. Sci..

[8] Zabkiewicz J.A., Pethiyagoda R., Forster W.A., van Leeuwen R., Moroney T.J., McCue S.W. (2020). Simulating spray droplet impaction outcomes: Comparison with experimental data. Pest. Manag. Sci..

[9] Tudi M., Daniel Ruan H., Wang L., Lyu J., Sadler R., Connell D., Chu C., Phung D.T. (2021). Agriculture Development, Pesticide Application and Its Impact on the Environment. Int. J. Environ. Res. Public. Health.

[10] Yao J., Zhi H., Shi Q., Zhang Y., Feng J., Liu J., Huang H., Xie X. (2023). Tannic Acid Interfacial Modification of Prochloraz Ethyl Cellulose Nanoparticles for Enhancing the Antimicrobial Effect and Biosafety of Fungicides. ACS Appl. Mater. Interfaces.

[11] Awwad M.M., Taha S.M., Khalil M.M.H., Salem A.M., Chovelon J.-M. (2023). The simultaneous degradation of prochloraz and tebuconazole in water with monitoring their degradation products using liquid chromatography-tandem mass spectrometry. Environ. Sci. Pollut. Res. Int..

[12] Zhao P., Cao L., Ma D., Zhou Z., Huang Q., Pan C. (2018). Translocation, distribution and degradation of prochloraz-loaded mesoporous silica nanoparticles in cucumber plants. Nanoscale.

[13] Dengjun W., Navid B.S., Andrew B., Richard Z., Endalkachew S.-D., Todd P.L., Kay T.H., Robert M.B., Markus F., Jason C.W. (2022). Nano-enabled pesticides for sustainable agriculture and global food security. Nat. Nanotechnol..

[14] Bueno V., Gao X., Abdul Rahim A., Wang P., Bayen S., Ghoshal S. (2022). Uptake and Translocation of a Silica Nanocarrier and an Encapsulated Organic Pesticide Following Foliar Application in Tomato Plants. Environ. Sci. Technol..

[15] Vijayakumar M.D., Surendhar G.J., Natrayan L., Patil P.P., Ram P.M.B., Paramasivam P. (2022). Evolution and Recent Scenario of Nanotechnology in Agriculture and Food Industries. J. Nanomater..

[16] Pan X., Guo X., Zhai T., Zhang D., Rao W., Cao F., Guan X. (2023). Nanobiopesticides in sustainable agriculture: Developments, challenges, and perspectives. Environ. Sci. Nano.

[17] Kah M., Beulke S., Tiede K., Hofmann T. (2013). Nanopesticides: State of Knowledge, Environmental Fate, and Exposure Modeling. Crit. Rev. Environ. Sci. Technol..

[18] Kah M., Hofmann T. (2014). Nanopesticide research: Current trends and future priorities. Environ. Int..

[19] Rehman A., Feng J., Qunyi T., Korma S.A., Assadpour E., Usman M., Han W., Jafari S.M. (2021). Pesticide-loaded colloidal nanodelivery systems; preparation, characterization, and applications. Adv. Colloid Interface Sci..

[20] Young M., Ozcan A., Myers M.E., Johnson E.G., Graham J.H., Santra S. (2017). Multimodal Generally Recognized as Safe ZnO/Nanocopper Composite: A Novel Antimicrobial Material for the Management of Citrus Phytopathogens. J. Agric. Food Chem..

[21] Maity D., Gupta U., Saha S. (2022). Biosynthesized metal oxide nanoparticles for sustainable agriculture: Next-generation nanotechnology for crop production, protection and management. Nanoscale.

[22] Mohapatra S., Bhakuni P., Barman S.R., Nayak B. (2024). RSM-CCD optimized hollow mesoporous silica nanospheres encapsulating sorafenib induce mitochondrial membrane potential mediated apoptotic cell death in non-small cell lung cancer. Microporous Mesoporous Mater..

[23] Šoltys M., Balouch M., Kašpar O., Lhotka M., Ulbrich P., Zadražil A., Kovačík P., Štĕpánek F. (2018). Evaluation of scale-up strategies for the batch synthesis of dense and hollow mesoporous silica microspheres. Chem. Eng. J..

[24] El-Sawy H.S., Al-Abd A.M., Ahmed T.A., El-Say K.M., Torchilin V.P. (2018). Stimuli-Responsive Nano-Architecture Drug-Delivery Systems to Solid Tumor Micromilieu: Past, Present, and Future Perspectives. ACS Nano.

[25] Camara M.C., Campos E.V.R., Monteiro R.A., do Espirito Santo Pereira A., de Freitas Proença P.L., Fraceto L.F. (2019). Development of stimuli-responsive nano-based pesticides: Emerging opportunities for agriculture. J. Nanobiotechnol..

[26] Gao Y., Liang Y., Dong H., Niu J., Tang J., Yang J., Tang G., Zhou Z., Tang R., Shi X. (2020). A Bioresponsive System Based on Mesoporous Organosilica Nanoparticles for Smart Delivery of Fungicide in Response to Pathogen Presence. ACS Sustain. Chem. Eng..

[27] Kaziem A.E., Gao Y., He S., Li J. (2017). Synthesis and Insecticidal Activity of Enzyme-Triggered Functionalized Hollow Mesoporous Silica for Controlled Release. J. Agric. Food Chem..

[28] Shrestha P.K., Chun Y.T., Chu D. (2015). A high-resolution optically addressed spatial light modulator based on ZnO nanoparticles. Light Sci. Appl..

[29] Sarma B., Sarma B.K. (2017). Fabrication of Ag/ZnO heterostructure and the role of surface coverage of ZnO microrods by Ag nanoparticles on the photophysical and photocatalytic properties of the metal-semiconductor system. Appl. Surf. Sci..

[30] Sobhani Z., Khalifeh R., Banizamani M., Rajabzadeh M. (2022). Water-soluble ZnO quantum dots modified by polyglycerol: The pH-sensitive and targeted fluorescent probe for delivery of an anticancer drug. J. Drug Delivery Sci. Technol..

[31] Singh P., Singh R.K., Kumar R. (2021). Journey of ZnO quantum dots from undoped to rare-earth and transition metal-doped and their applications. RSC Adv..

[32] Wang W., Wang Y., Wang Y., Gong H., Zhu H., Liu M. (2019). Redox/pH dual stimuli-responsive ZnO QDs-gated mesoporous silica nanoparticles as carriers in cancer therapy. IET Nanobiotechnol..

[33] Zhao W.-B., Du M.-R., Liu K.-K., Zhou R., Ma R.-N., Jiao Z., Zhao Q., Shan C.-X. (2020). Hydrophilic ZnO Nanoparticles@Calcium Alginate Composite for Water Purification. ACS Appl. Mater. Interfaces.

[34] Qiu J., Chen Y., Liu Z., Wen H., Jiang N., Shi H., Kou Y. (2023). The application of zinc oxide nanoparticles: An effective strategy to protect rice from rice blast and abiotic stresses. Environ. Pollut..

[35] Liang Y., Duan Y., Fan C., Dong H., Yang J., Tang J., Tang G., Wang W., Jiang N., Cao Y. (2019). Preparation of kasugamycin conjugation based on ZnO quantum dots for improving its effective utilization. Chem. Eng. J..

[36] Xie Q., Lu H., Wang X., Zhang Y., Zhou N. (2021). Functionalized hollow mesoporous silica for detection of Staphylococcus aureus and sterilization. J. Environ. Chem. Eng..

[37] Chen W., Cheng C.-A., Cosco E.D., Ramakrishnan S., Lingg J.G.P., Bruns O.T., Zink J.I., Sletten E.M. (2019). Shortwave Infrared Imaging with J-Aggregates Stabilized in Hollow Mesoporous Silica Nanoparticles. J. Am. Chem. Soc..

[38] Zhao N., Cai R., Zhang Y., Wang X., Zhou N. (2022). A pH-Gated Functionalized Hollow Mesoporous Silica Delivery System for Photodynamic Sterilization in Staphylococcus aureus Biofilm. Materials.

[39] Chen M., Hu J., Bian C., Zhu C., Chen C., Guo Z., Zhang Z., Agyekum G.A., Zhang Z., Cao X. (2020). pH-Responsive and Biodegradable ZnO-Capped Mesoporous Silica Composite Nanoparticles for Drug Delivery. Materials.

[40] Moyen E., Kim J.H., Kim J., Jang J. (2020). ZnO Nanoparticles for Quantum-Dot-Based Light-Emitting Diodes. ACS Appl. Nano Mater..

[41] Shi L., Liang Q., Zang Q., Lv Z., Meng X., Feng J. (2022). Construction of Prochloraz-Loaded Hollow Mesoporous Silica Nanoparticles Coated with Metal–Phenolic Networks for Precise Release and Improved Biosafety of Pesticides. Nanomaterials.

[42] Wu L., Pan H., Huang W., Hu Z., Wang M., Zhang F. (2022). pH and Redox Dual-Responsive Mesoporous Silica Nanoparticle as Nanovehicle for Improving Fungicidal Efficiency. Materials.

[43] Wang C.-Y., Lou X.-Y., Cai Z., Zhang M.-Z., Jia C., Qin J.-C., Yang Y.-W. (2021). Supramolecular Nanoplatform Based on Mesoporous Silica Nanocarriers and Pillararene Nanogates for Fungus Control. ACS Appl. Mater. Interfaces.

[44] Liang W., Xie Z., Cheng J., Xiao D., Xiong Q., Wang Q., Zhao J., Gui W. (2021). A Light-Triggered pH-Responsive Metal–Organic Framework for Smart Delivery of Fungicide to Control Sclerotinia Diseases of Oilseed Rape. ACS Nano.

[45] Ma Y., Li L., Zhao R., Sun Z., Wang Y., Yu M., Pan S., Guo X., Xu Y., Wang H. (2023). Nanoencapsulation-based fabrication of eco-friendly pH-responsive pyraclostrobin formulations with enhanced photostability and adhesion to leaves. J. Environ. Chem. Eng..

[46] Abdelrahman T.M., Qin X., Li D., Senosy I.A., Mmby M., Wan H., Li J., He S. (2021). Pectinase-responsive carriers based on mesoporous silica nanoparticles for improving the translocation and fungicidal activity of prochloraz in rice plants. Chem. Eng. J..

[47] Muhammad F., Guo M., Qi W., Sun F., Wang A., Guo Y., Zhu G. (2011). pH-Triggered controlled drug release from mesoporous silica nanoparticles via intracelluar dissolution of ZnO nanolids. J. Am. Chem. Soc..

[48] Askarizadeh M., Esfandiari N., Honarvar B., Sajadian S.A., Azdarpour A. (2023). Kinetic Modeling to Explain the Release of Medicine from Drug Delivery Systems. ChemBioEng Rev..

[49] Pooresmaeil M., Namazi H. (2020). Facile preparation of pH-sensitive chitosan microspheres for delivery of curcumin; characterization, drug release kinetics and evaluation of anticancer activity. Int. J. Biol. Macromol..

[50] Ritger P.L., Peppas N.A. (1987). A simple equation for description of solute release II. Fickian and anomalous release from swellable devices. J. Control. Release.

[51] Rani S., Kumari N., Sharma V. (2022). Uptake, translocation, transformation and physiological effects of nanoparticles in plants. Arch. Agron. Soil. Sci..

[52] Afzal S., Aftab T., Singh N.K. (2022). Impact of Zinc Oxide and Iron Oxide Nanoparticles on Uptake, Translocation, and Physiological Effects in *Oryza sativa* L. J. Plant Growth Regul..

