# Supplementary material for: pH-Responsive Pesticide-Loaded Hollow Mesoporous Silica Nanoparticles with ZnO Quantum Dots as a Gatekeeper for Control of Rice Blast Disease
Source: Materials (Basel). 2024 Mar 14;17(6):1344. doi: 10.3390/ma17061344 (PMC10972096; doi:10.3390/ma17061344)
Supplement: Supplementary file 1 [file materials-17-01344-s001.zip › materials-2896062-supplementary.pdf]

Supporting Information

pH-Responsive Pesticide-Loaded Hollow Mesoporous Silica Nanoparticles with ZnO Quantum Dots as a Gatekeeper for Control of Rice Blast Disease

Yi Zhao, Yanning Zhang, Yilin Yan, Zunyao Huang, Yuting Zhang, Xiaoli Wang* and Nandi Zhou*

The Key Laboratory of Carbohydrate Chemistry and Biotechnology, Ministry of Education, School of Biotechnology, Jiangnan University, Wuxi 214122, China

*Corresponding authors. E-mail addresses: xiaoliwang@jiangnan.edu.cn (X. Wang); zhounandi@jiangnan.edu.cn (N. Zhou)

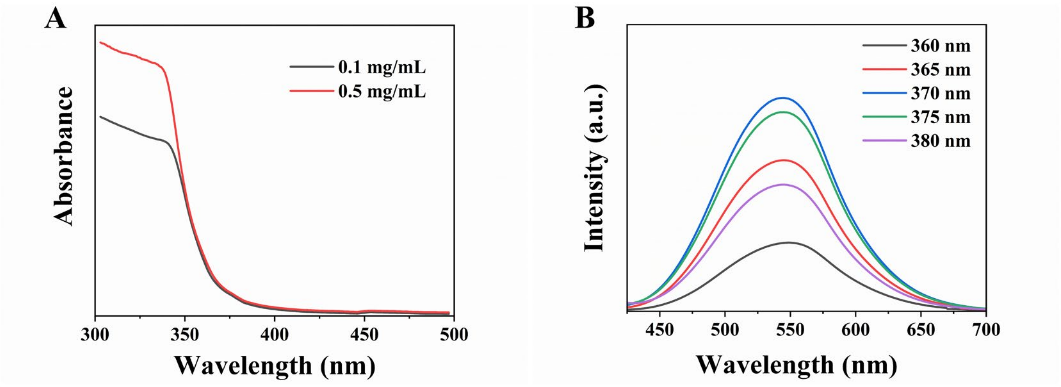

Figure S1. (A) UV-vis absorption spectra of ZnO QDs at different concentrations. (B) Fluorescence spectra of ZnO QDs at different excitation wavelength.

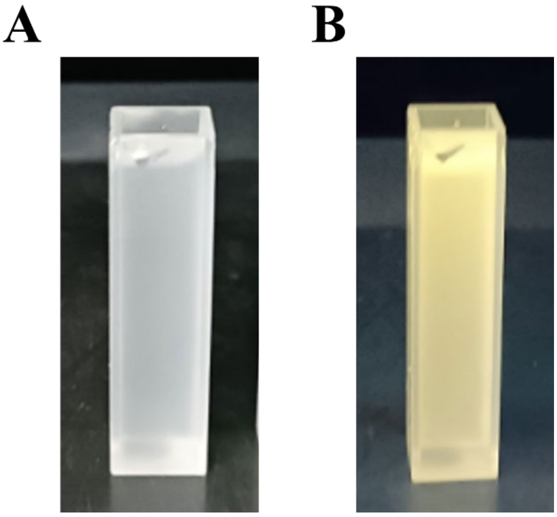

Figure S2. Digital images of ZnO QDs under daylight (A) and 365 nm UV lamp (B).

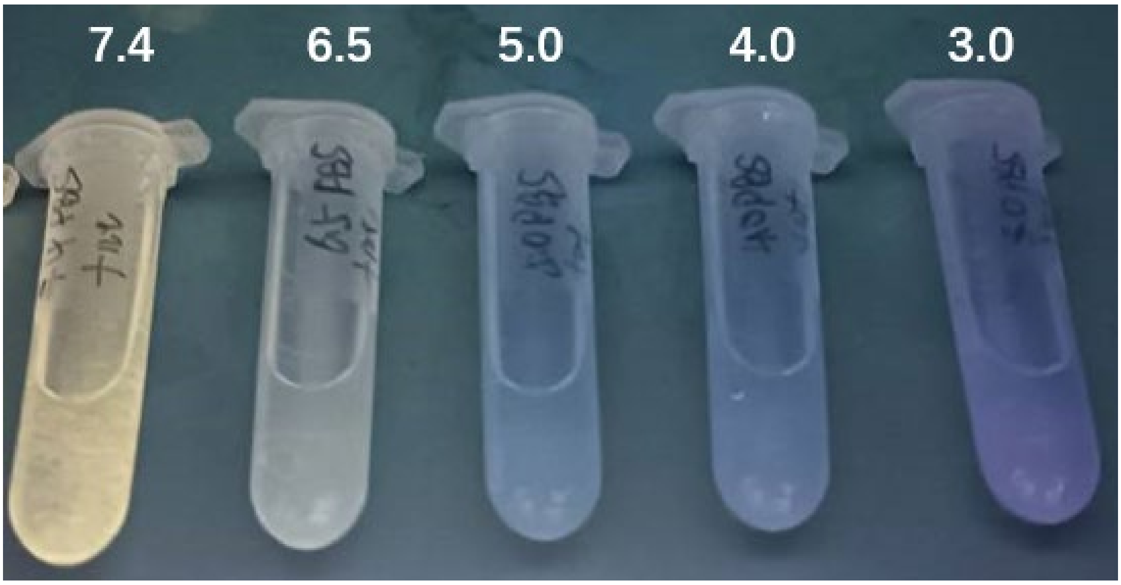

Figure S3. Dissolution of ZnO QDs in PBS at different pH.

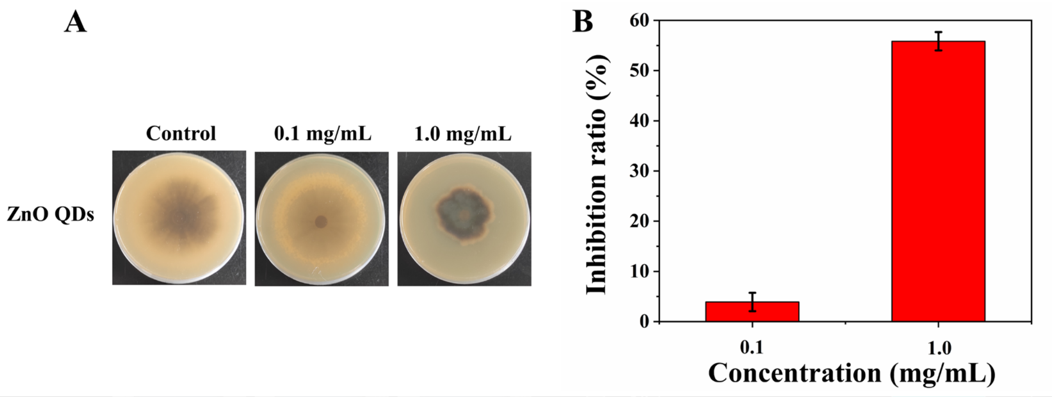

Figure S4. (A) Images of *M. oryzae* treated with different concentration of ZnO QDs for 13 days. (B) Inhibition ratio of *M. oryzae* treated with different concentration of ZnO QDs for 13 days.

Table S1. Release kinetic equations of HMSNs@Pro at different pH.

pH	Models	Fitting equation	R^2
	Zero-order	$y=1.20\ x+21.68$	0.5957
5.0	Higuchi	$y=10.35\ x^{1/2}+6.33$	0.8755
	Ritger-Peppas	$y=17.93\ x^{0.36}$	0.9237
	Zero-order	$y=1.31\ x+22.61$	0.6077
6.5	Higuchi	$y=11.25\ x^{1/2}+6.05$	0.9151
	Ritger-Peppas	$y=18.59\ x^{0.37}$	0.9237
	Zero-order	$y=0.90\ x+29.13$	0.3273
7.4	Higuchi	$y=8.74\ x^{1/2}+14.88$	0.6694
	Ritger-Peppas	$y=25.99\ x^{0.25}$	0.8448

Table S2. Release kinetic equations of HMSNs@Pro@ZnO QDs at different pH.

pH	Models	Fitting equation	R^2
	Zero-order	$y=0.93\ x+13.63$	0.6756
5.0	Higuchi	$y=7.80\ x^{1/2}+2.41$	0.9152
	Ritger-Peppas	$y=10.96\ x^{0.41}$	0.9357
	Zero-order	$y=0.64\ x+9.13$	0.7424
6.5	Higuchi	$y=5.26\ x^{1/2}+1.74$	0.9496
	Ritger-Peppas	$y=7.28\ x^{0.42}$	0.9637
	Zero-order	$y=0.27\ x+8.68$	0.4102
7.4	Higuchi	$y=2.49\ x^{1/2}+4.74$	0.7425
	Ritger-Peppas	$y=7.97\ x^{0.23}$	0.9306

Table S3. Fungicidal activity of Pro technical and HMSNs@Pro@ZnO QDs against *M. oryzae*.

Group	Regression equation	EC_{50}	R^2	95% Confidence limits
Pro technical	y=2.292+2.171x	0.088	0.987	0.076-0.103
HMSNs@Pro@ZnO QDs	y=2.677+2.339x	0.072	0.979	0.063-0.082

Table S4. Effects of Pro technical, HMSNs@Pro@ZnO QDs and 0.1% tween-80 on the lesion development by *M. oryzae* on rice leaves.

Group	Concentration (μg/mL)	Lesion length (cm^2)	Control efficacy (%)
Pro technical	100	0.454	86.72
HMSNs@Pro@ZnO QDs	100	0.362	89.40
Control	-	3.416	-
